# Facile synthesis of phosphorus and nitrogen co-doped carbon dots with excellent fluorescence emission towards cellular imaging†

**DOI:** 10.1039/d3ra03361a

**Published:** 2023-07-12

**Authors:** Fang-Jun Cao, Xiang Hou, Kai-Feng Wang, Tie-Zhi Jin, Hui Feng

**Affiliations:** a Shaanxi Key Laboratory of Qinling Ecological Security, Shaanxi Institute of Zoology Xi'an Shaanxi 710072 P. R. China jintiezhi@xab.ac.cn fenghui84@126.com

## Abstract

Fluorescent carbon nanomaterials have attracted increasing attention owing to their unique photoluminescence properties, good biocompatibility and low toxicity in bioimaging as well as biosensing. Heteroatom doping is usually used to improve photoluminescence properties by tuning the functional groups and the particle size domain effect, thus leading to redshifted emission. Here, we report a straightforward strategy for the fabrication of a mixture of fluorescent phosphorus and nitrogen carbon nanodots (P,N-CDs) followed by separating two kinds of fluorescent fractions based on their different negative charges. Such a one-pot hydrothermal method using formamide, urea and hydroxyethylidene diphosphonic acid as the precursor yields fluorescent P,N-CDs. Specifically, blue-emitting CDs (bCDs) and green-emitting CDs (gCDs) were separated by using column chromatography. The quantum yields of bCDs and gCDs were 20.33% and 1.92%, respectively. And the fluorescence lifetimes of bCDs and gCDs were 6.194 ns and 2.09 ns, respectively. What is more, the resultant P,N-CDs exhibited low toxicity and excellent biocompatibility. Confocal fluorescence microscopy images were obtained successfully, suggesting that P,N-CDs have excellent cell membrane permeability and cellular imaging. This work provides a promising fluorescent carbon nanomaterial with tunable emission as a probe for versatile applications in bioimaging, sensing and drug delivery.

## Abstract 2

The fluorescent P,N-CDs were prepared by the facile solvothermal decomposition of urea and HEDP in formamide. The resultant P,N-CDs exhibit low toxicity, excellent biocompatibility and bioimaging.

## Graphical abstract

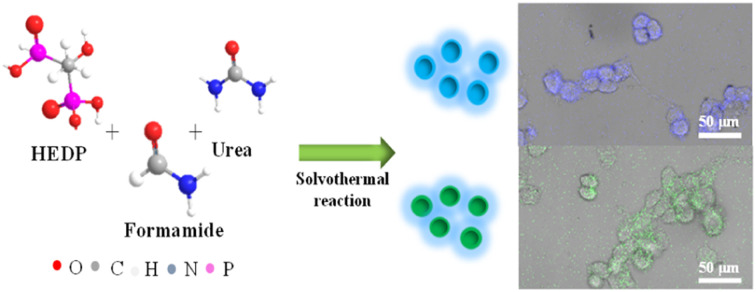

## Introduction

Carbon dots (CDs), as promising nanomaterials, have attracted the interest of researchers because of their fascinating optical and biological properties, such as photoluminescence, biocompatibility, and electrochemistry.^1,2^ Compared to other traditional fluorescent probes, such as organic dyes (*e.g.*, rhodamine, porphyrin and cyanine derivatives) and nanomaterials (*e.g.*, quantum dots, silicon nanoparticles and gold nanoclusters),^3,4^ photoluminescent CDs exhibit great photostability, satisfactory Stokes shift, excellent solubility and extraordinary biocompatibility and have attracted broad attention as new probes for biological imaging *in vitro* and *in vivo*.^5–8^ In fluorescent carbon quantum dots, the fluorescence emission has been ascribed to the “quantum effect”, in which the particle scale of the CDs results in coalescence of energy levels enabling excitation of elections to the conducting band from the valence band in discreet energies.^9^ In addition, many studies have demonstrated that the fluorescence properties of CDs are closely related to the particle surface. In particular, surface defects are responsible for light absorbance in different colors.^10^ Therefore, it is necessary to understand the relationship between the structure and the luminescence mechanism of CDs, so as to guide their functional modification and endow them with specific fluorescence properties in the biomedical field.

In recent years, various “top-down” and “bottom-up” approaches have been developed to prepare and tune the optical properties of CDs.^11–15^ At present, several main luminescence mechanisms have been reported, including carbon nuclear state, surface state and molecular state. First, in the emission of the “carbon nuclear state”, a large amount of sp^2^ carbon forms a conjugated π domain, and the radiation is caused by the band gap transition in the π region. Based on the quantum confinement effect, the band gap transition and characteristic fluorescence emission are affected by the quantum size dependence of CDs. This luminescence mechanism is applicable to explain the luminescence of CDs with lattice structures or high graphitization.^16^ Li's group prepared four CDs with different sizes by an electrochemical method under alkaline conditions and achieved fluorescence emission behavior from the 350–800 nm ultraviolet region to the near-infrared region.^17^ In addition, various functional groups on the surface of CDs have different energy levels, which may produce different emission traps. The oxygen-containing functional group as the emission capture center of the exciton can regulate the fluorescence emission properties of CDs. Ding's group prepared and isolated multicolor CDs with tunable photoluminescence and quantum yields up to 35%.^18^ The redshift of the fluorescence emission wavelength is attributed to the gradual reduction of its band gap, which is due to the increase in the incorporation of different oxygen-containing functional groups in its surface structure. During the synthesis of CDs, molecular precursors possess active functional groups (such as carboxyl and amino groups) in the structure, carbon precursors can easily react with each other, further condense, polymerize and carbonize, which may produce small fluorescent molecules or oligomers that are connected to the interior and surface of the carbon skeleton, giving CDs bright emission characteristics.^19^ Song's group prepared CDs with citric acid (CA) and ethylenediamine (EDA) and studied their luminescence mechanism.^20^ They constructed imidazo[1,2-*a*]pyridine-7 through a small molecule organic synthesis reaction-carboxylic acid (IPCA) and a series of characterizations confirmed that CDs contain molecular IPCA. The process of synthesizing this molecular state from Ca and EDA at 140 °C produces stronger polymerization and carbonization reactions at higher temperatures. Thus, the study on an efficient method for the synthesis and luminescence mechanism of double heteroatom-doped CDs is highly necessary.

In this work, we developed a facile strategy for the fabrication of highly fluorescent phosphorus and nitrogen carbon nanodots and demonstrated their applications for cellular imaging. Hydroxyethylidene diphosphonic acid (HEDP) and formamide with abundant phosphorus and nitrogen components were chosen as the precursors. Accordingly, a mixture of P- and N-embedding CDs was obtained through a one-pot hydrothermal treatment. The separation of the mixture solution allows individual fluorescent fractions to be obtained by column chromatography. The existence of graphitic nitrogen represented an intrinsic variable allowing to gain the red-shift in P,N-CDs along with already applied strategies regulating types of surface molecular fluorophores. The as-prepared P,N-CDs showed remarkable photoluminescence (PL) features with excellent photostability. More importantly, the two highly biocompatible P,N-CDs exhibited high cell penetrability, low toxicity and excellent cell imaging performance. This work provides a facile and general method to fabricate fluorescence carbon dots for use as drug carriers in therapeutic applications.

## Experimental and characterization

### Chemicals

The formamide, urea, hydroxyethylidene diphosphonic acid (HEDP) and dimethyl sulfoxide (DMSO) were obtained from Aladdin Reagent Co., Ltd (Shanghai, China). Hydrochloric acid was purchased from Shanghai Yuanye Biotechnology Co., Ltd (Shanghai, China). All reagents were analytical grade. McCoy's 5A medium, MEM medium, fetal bovine serum (FBS), trypsin, 3-(4,5-dimethylthiazol-2-yl)-2,5-diphenyl-tetrazolium bromide (MTT) were bought from GIBCO BRL Co. (New York, USA). Deionized water was used in the entire process of the experiments.

### Synthesis of P,N-CDs

**Fig. 1:**
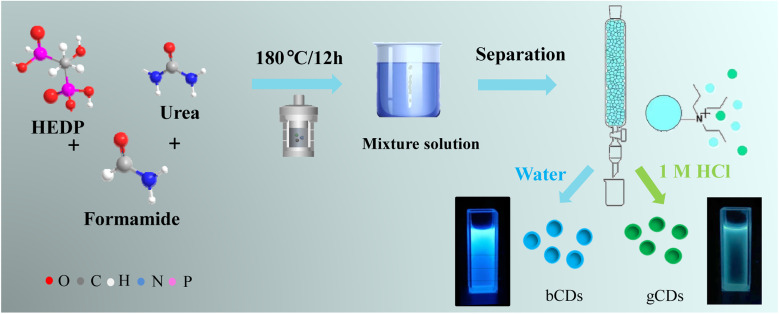
Reaction scheme used for the synthesis of a P,N-CD mixture and schematic diagram of anion-exchange separation process.

The mixture of P,N-CDs was synthetized according to the following procedures, as shown in Fig. 1. HEDP (100 mg) and urea (100 mg) were dissolved by mild ultrasonication in 10 mL of formamide. The clear solution was subsequently transferred into a 50 mL Teflon-lined stainless-steel autoclave and put into an oven heated at 180 °C. After a reaction time of 12 h, the autoclave was cooled to room temperature.

Furthermore, the obtained mixture solution was immediately used for separation. The glass column filled with the DOWEX 1 × 8 chloride form (100–200 mesh) (30 mL) was equilibrated with 0.5 M HCl, followed by washing with deionized water repeatedly to a neutral pH. The prepared mixture of CDs (1 mL) was carefully loaded on the column in which the unbound fractions were washed with deionized water. Under irradiation with a 365 nm UV lamp, the fluorescence of the eluted fractions was controlled, and the blue luminescence material (bCDs) was separated by deionized water. After elution with a 1.0 M HCl, the green-emitting CDs (gCDs) were collected. The two types of separated fractions were filtered in a 2 kDa cut-off dialysis membrane against deionized water.

### Quantum yields measurements

The fluorescence quantum yield of as-synthetized P,N-CDs was measured using fluorescence spectrophotometer at room temperature, the dilute solution of CDs and reference solvent were measured. Based on the integrated fluorescence intensity obtained under the excitation wavelength of 374 nm and the absorbance of incident light obtained at that excitation wavelength, the quantum yield was calculated.

### Determination of fluorescence lifetime

The fluorescence lifetime of P,N-CDs was measured using a fluorescence spectrometer, with excitation and emission wavelengths of 375 nm and 500 nm, respectively.

### Effect of different ionic strength on fluorescence

200 μL P,N-CDs solution (8 mg mL^−1^) was added to 2 mL of sodium chloride buffer solution with different concentrations, so that the concentration of sodium chloride in the solution is 0.2, 0.6, and 1 mol L^−1^. Then, using a fluorescence spectrophotometer at an excitation wavelength of 374 nm (*λ*_ex_) to record the fluorescence emission spectrum.

### Determination of pH on sample stability

The P,N-CDs solution with an UV absorbance of 0.1–0.2 was prepared, so that the concentration of sodium chloride buffer solution with pH = 3, 7, and 11, and the UV absorption spectra of each mixed solution was measured, respectively.

### Determination of ionic strength on sample stability

The P,N-CDs solution with an UV absorbance of 0.1–0.2 was prepared, so that the concentration of sodium chloride in the solution is 0.2, 0.6, and 1 mol L^−1^. The UV absorption spectra of each mixed solution was measured.

### Cell culture and MTT assay

The inhibition rate and growth curve were tested using the MTT assay. Briefly, 5.0 × 10^3^ cells (U20S cells and 143B cells) per well were seeded into 96-well cell culture plates and then incubated with a gradient dose of the tested P,N-CD solution (150 μL) for 24 h. Subsequently, 30 μl MTT solution (5 mg mL^−1^ in PBS) was added to the plate, and the cells were cultured for an additional 4 h. After discarding the supernatant, 150 μL DMSO was used to dissolve the formed formazan crystals until the solution turned purple. The absorbance was then determined at 570 nm using a microplate reader (Bio-Rad 680). The related growth inhibition rates (GIRs) were measured by the equation GIRs = (*A*_c_ − *A*_t_)/*A*_c_ × 100, among which *A*_c_ and *A*_t_ represent the absorbances in the control and treated groups, respectively.

### Confocal microscopy imaging

Cells were seeded into 35 mm special laser confocal culture dish (10^4^ cells per cell culture dish) in DMEM overnight and co-incubated with 0.08 mg mL^−1^ of P,N-CDs for 2 hours at 37 °C. Then, the cells were washed twice with PBS buffer to remove the residual P,N-CDs in the culture medium. The representative fields were observed and photographed with a confocal microscope (Leica, Wetzlar, Germany).

### Characterization techniques

The microscopic images and structure were measured by Transmission Electron Microscope (TEM) with an aberration-corrected HAADF-STEM instrument at 300 kV combined with SEM at 15 kV. The size of the nanoparticles and histograms were measured by ImageJ software by optional counting of 1000 particles. The UV-vis spectra were determined by a UV-vis spectrometer. The surface elements and chemical states of the samples were investigated by X-rayphotoelectron spectroscopy technology (XPS). The phase and morphology of the as-separated P,N-CDs were determined by X-ray diffraction (XRD). The groups and chemical bond properties of the P,N-CDs were characterized by an infrared spectrometer. Raman spectrum analysis was conducted on a Raman spectrometer. The fluorescence properties of P,N-CDs were tested by an fluorescence spectrophotometer with excitation wavelengths from 380 nm to 450 nm. The relevant instrument equipment and models are listed in Table S1†.

## Results and discussion

As presented in Fig. 1, the mixture solution was prepared using HEDP and urea dissolved in formamide as a carbon precursor through a solvothermal method. The formation mechanisms of our P,N-CDs could be attributed to the hydrolysis, dehydration and fragmentation of small molecules. Then, the as-synthesized mixture of CDs was isolated through a prepreparative anion-exchange column (Dowex 1 × 8 chloride form) to obtain fractions of CDs exhibiting blue and green fluorescence alone. The factions of blue fluorescent CDs (bCDs) showed relatively neutral charge and were first separated from the column using just deionized water, whereas green fluorescent CDs (gCDs) with the negative charge were separated and eluted with a 1.0 M solution of HCl with negative charge.

**Fig. 2:**
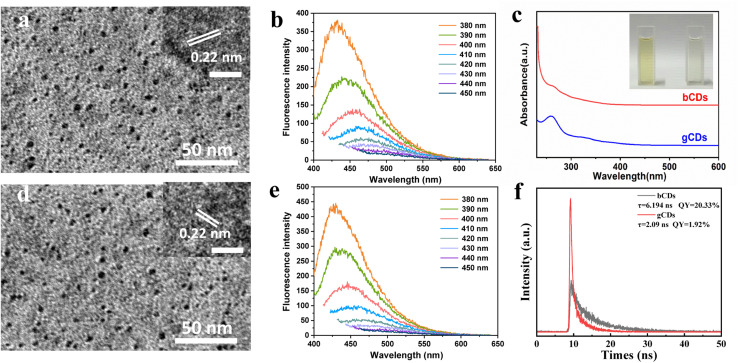
Structure characterization diagrams of bCDs and gCDs. (a) TEM image and HRTEM of bCDs; (b) photoluminescence emission spectra of the bCDs excited at different excitation wavelengths; (c) UV-vis absorption of the bCDs and gCDs; (d) TEM image and HRTEM of the separated fractions of gCDs; (e) photoluminescence emission spectra of the gCDs excited at different excitation wavelengths; (f) fluorescence lifetime of the separated fractions of bCDs and gCDs.

The structural morphology of the synthesized CDs was initially investigated by transmission electron microscopy (TEM). Fig. S1† depicts TEM image and the size distribution and morphology of the bCDs and gCDs. The prepared bCDs and gCDs are well-dispersed spherical structures with an average size of 2–4 nm. The high-resolution TEM (HRTEM) image of the bCDs and gCDs indicated that clear lattice fringes with spacings of 0.22 nm could be indexed to (100) graphitic carbon, respectively (Fig. 2a and d). The average sizes of the bCDs and gCDs were approximately 2 nm. Furthermore, in light of the comparison of HRTEM images of bCDs and gCDs (see insets in Fig. 2a and d), the size difference was not statistically significant. The X-ray diffraction (XRD) patterns of the bCDs and gCDs (Fig. S2†) showed a broad peak indicating that the CDs have the amorphous nature.^21^ In addition, by ultraviolet analysis, the absorption spectra of the two eluted fractions exhibited a well-resolved π–π* transition in the spectrum ranging from 200 nm to 250 nm and an obvious n–π* transition at 340 nm attributed to the typical characteristics of nitrogen-doped CDs (Fig. 2c).^22–24^ Meanwhile, according to the color under the UV lamp, bCDs and gCDs had emission maxima at 460 nm and 510 nm, respectively. The fluorescence strength of traditional organic dyes universally decayed quickly under continuous illumination. However, the fluorescence strength of the separated fractions had no decay after several hours of UV irradiation, indicating their good photobleaching resistance.^25^ It is worth noting that the as-eluted bCDs and gCDs had no fluorescence attenuation, even if the preservation time lasted over several weeks, indicating their significant photostability.

The bCDs and gCDs possessed excellent fluorescence properties depending on the wavelength of incident light. The bCDs and gCDs displayed excellent fluorescence properties, which were correlated with the wavelength of incident light. The PL curves of the CDs were determined at various excitation wavelengths, as shown in Fig. 2b and e. The as-separated bCDs and gCDs possessed remarkable excitation wavelength-dependent emission spectra, so the bCDs and gCDs could be used for imaging applications under different excitation wavelengths. The spectral analysis indicated that the excitation wavelength shifted from 400 nm to 600 nm, resulting in the PL peak gradually changing from 380 (orange) to 450 nm (dark blue). The excitation wavelength of the bCDs mostly relied on the size dimension and the distinct emissive trap sites of the bCDs, in contrast, had an effect on their fluorescence.^26^ Similar phenomena were also found in the gCDs. The possible luminescence mechanism of these separated fractions can be attributed to the carbon nuclear state, surface state and molecular state. The obviously reducing intensity of the G-band at 1590 cm^−1^ and rising intensity of the D-band at 1340 cm^−1^ from blue to green CDs. This feature can be analysed by a higher number of structurally contained graphitic nitrogen atoms to the sp^2^ scaffold.^27–29^ Individual samples were further analysed by Fourier transform infrared (FT-IR) spectroscopy to identify differences responsible for fluorescence emission. Fig. S3† displays the Fourier transform infrared (FTIR) spectra of the samples. FT-IR analysis confirmed an obvious aromatic structure at 1600 cm^−1^ typical for C=C bonds in all fractions. Similar to the surface-related C–N bonds at 1017 cm^−1^, carboxylic C=O bonds at 1710 cm^−1^ were significant in both the bCD and gCD samples.^30^ The spectra of the bCDs and rCDs displayed no obvious peaks at 1360 and 1650 cm^−1^ typical for C–N= bonds and C=N bonds. The above results showed that the CDs were mostly rich in nitrogen, oxygen and phosphorus. Among of them, oxygen was originated from hydroxyl, carbonyl and carboxylic acid groups, nitrogen and phosphorus were derived from urea, formamide and HEDP.

**Fig. 3:**
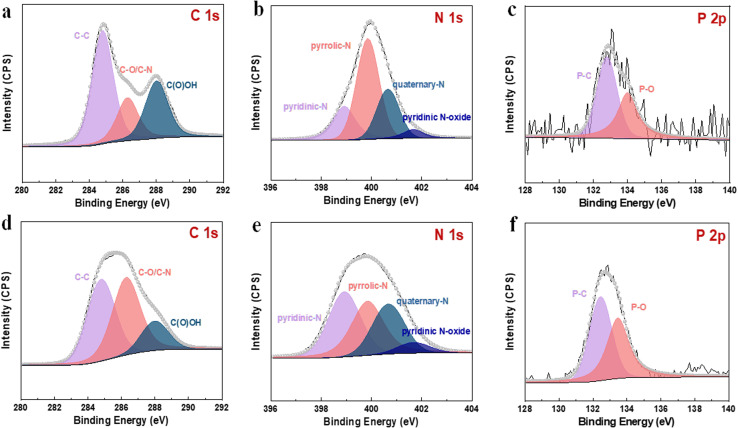
High-resolution XPS spectra of the as-prepared samples. (a–c) High-resolution C 1s, N 1s and P 2p XPS spectra for bCDs. (d–f) High-resolution C 1s, N 1s and P 2p XPS spectra for gCDs.

To further evaluate the fluorescence performance of gained bCDs and gCDs solution, fluorescence spectrophotometer were used to investigated their fluorescence quantum yields. The fluorescence quantum yields of bCDs and gCDs were 20.33% and 1.92%, respectively (Fig. 2f). In addition, we investigated the fluorescence lifetime of gained CDs with maximum excitation and emission wavelengths at 375 nm and 500 nm, respectively. The fluorescence lifetimes of bCDs and gCDs were about 6.194 ns and 2.09 ns, respectively. The results indicated that the excited fluorescence intensity of CDs samples eluted with acid showed fast decay and relatively short lifespan. To assess the composition of the as-synthesized P,N-CDs, XPS measurements were further conducted. Table S2† summarizes the elemental analysis of P,N-CDs. For bCDs, the doping concentrations of P and O are approximately 1.26 and 33.76%, respectively. Fig. S4a† shows the survey XPS scan of the bCDs sample. The binding energy peaks at 131.5, 285.0, 398.2, and 531.0 eV indicate the presence of P, C, N and O. Fig. 3a displays the high-resolution C 1s spectrum, which can be deconvoluted into six peaks at 284.8, 286.29 and 288.02 eV, representing C 1s states in C–C, C–N and C(O)OH, respectively.^31,32^ The N 1s spectrum (Fig. 3b) shows four peaks at 398.7, 398.92, 399.84 and 400.67 eV, which are associated with pyridinic-N-oxide, pyridinic-N, pyrrolic-N and quaternary-N, respectively.^33^Fig. 3c shows the high-resolution P 2p peak with obvious P–C bands (132.5 eV) and P–O bands (133.5 eV), indicating the presence of phosphorus species.^34^ Fig. S4b† and 3e–f shows the survey XPS scan of the gCDs sample and high-resolution C 1s, N 1s and P 2p XPS spectra, respectively. Similar absorption patterns can be observed in gCDs sample. The high-resolution P 2p XPS spectra showed a number of phosphorous groups in CDs (Fig. 3c and f). By analysing the high-resolution N 1s XPS spectra, it was confirmed that the most vital parameter causing the shift from blue to green photoluminescence was the growing tendency of graphitic nitrogen in terms of quantity located at approximately 401.6–401.3 eV (Fig. 3b and e). Nitrogen from surface amide groups (399.7–400.1 eV) was present in the bCDs and gCDs. These findings are consistent with the specific existence of graphitic nitrogen in full-color fluorescent CDs.^35^ This fact indicated that the main factor of graphitic nitrogen as an “intrinsic parameter”, resulting in the redshift of photoluminescence in the as-separated CDs. Besides, Raman spectrum showed that *A*_D_/*A*_G_ was about 1.065 (Fig. S5†), further suggesting a low graphitic level for the bCDs and gCDs.

In addition, the influence of sodium chloride ions with different concentrations on the fluorescence of CDs was tested by fluorescence emission spectrum. As shown in Fig. S6,† as the concentration of sodium chloride ions is up to 1.0 mol L^−1^, the fluorescence effect on both CDs is relatively small. Especially, after the UV absorption spectra of each mixed P,N-CDs solution was measured in hydrochloric acid solutions of different PH and sodium chloride ionic solution of different concentrations, it was suggested that the as-synthesized P,N-CDs solution show relatively strong stability, and PH value and the concentration of sodium chloride have almost no effect on their stability (Fig. S7 and S8†). Based on the discussion above, it was suggested that the as-synthesized P,N-CDs solution showed relatively strong stability.

**Fig. 4:**
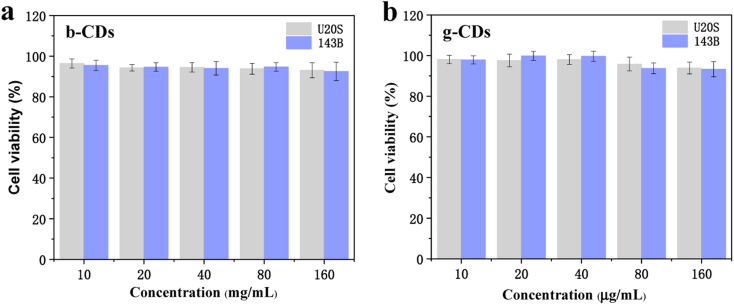
(a and b) The cytotoxic activities of bCDs and gCDs on U20S and 143B cells at 0–160 μg mL^−1^ (from left to right).

As a new fluorescent sensor, the application of in bioimaging was further explored *in vitro*. As shown in Fig. 4, the relative survival rate of U20S and 143B cells exposed to P,N-CDs was measured by the MTT method to evaluate the cytotoxicity of N,P-CDs. Based on the concentration screening test, the concentrations of the bCDs were set as 10, 20, 40, 80, and 160 μg mL^−1^ for U20S and 143B cells. When the concentration of bCDs increased to 160 μg mL^−1^, more than 90% of the cells could still survive. Similar to bCDs, the viability of gCD remained greater than 92.6%, demonstrating the low toxicity of the P,N-CDs. Hence, the prepared P,N-CDs with high stability and low cytotoxicity can be used for potential fluorescent cellular imaging.

**Fig. 5:**
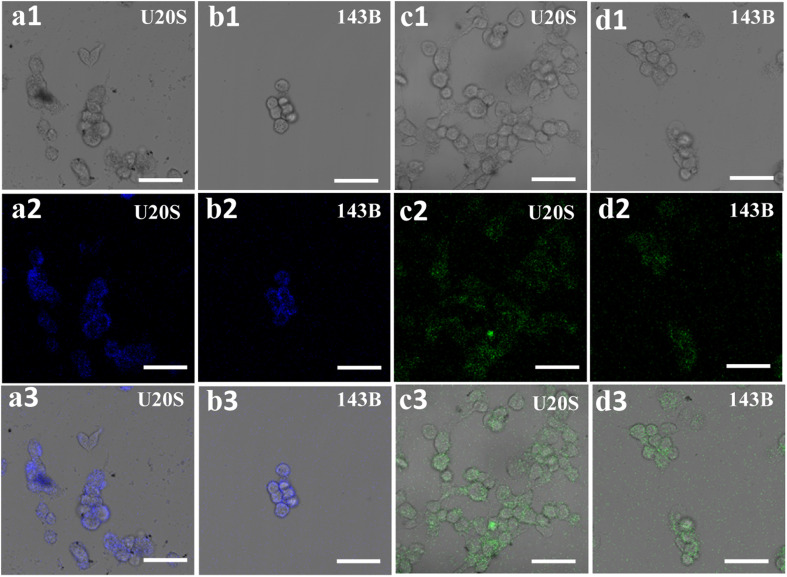
Bright (a1–d1) and fluorescence (a2–d2) and merged (a3–d3) images of 143B cells incubated with bCDs and gCDs obtained using laser scanning confocal microscopy (LSCM) (SP8) (from left to right). Scale bars: 50 μm.

To explore the potential cellular imaging of the sample for living cells, U20S and 143B cells were exposed to bCD and gCD aqueous solutions, as shown in Fig. 5. For U20S cells, P,N-bCDs irradiated by 405 nm laser wavelengths were mainly located in the cytoplasm, and cells incubated with P,N-CDs showed multicolor excitation-dependent fluorescence (Fig. S9†). The as-separated bCDs could easily penetrate the cytoplasm as well as the cell membrane. In addition, the cells incubated with gCDs display green emissions at a *λ*_Em_ of 488 nm. In particular, the bright blue and green fluorescence was almost distributed on the cell membrane, indicating that the two types of fractions had difficulty in infiltrating into the inner nuclei (Fig. 5a and c). A similar phenomenon can be observed in 143B cells (Fig. 5b and d), and the cells incubated with P,N-CDs maintained good morphology, which means that P,N-CDs are biocompatible and have minimal cytotoxicity to cells.^36,37^ Therefore, the prepared P,N-CDs have excellent biocompatibility and good fluorescence performance, suggesting that the potential biomedical applications in early diagnosis of tumors and other diseases.

## Conclusion

In summary, a mixture of fluorescent P,N-CDs had been prepared by facile solvothermal decomposition of HEDP and urea in formamide. The individual fluorescent fractions were separated by column chromatography based on the differences in surface charge. The as-prepared bCDs and gCDs had average grain diameter with 2–4 nm. With increasing excitation wavelength, the P,N-CDs had excitation wavelength dependence. And the characters of fluorescence quantum yield and lifetime were also investigated. The fluorescence quantum yields of bCDs and gCDs were about 20.33% and 1.92% and the fluorescence lifetimes of bCDs and gCDs were about 6.194 ns and 2.09 ns, respectively. What is more, the osteosarcoma cells incubated with two fluorescent CDs could emit bright blue/green fluorescence under both excitation light sources. Therefore, the as-prepared CDs demonstrated as luminescence probe with excellent PL performance, including high structural stability, bright luminescence, excitation wavelength-dependent emission, stable fluorescence intensity, biocompatibility, and high photostability. The present work provides a general and facile way to prepare blue/green fluorescent CDs, offering great application value in biomedical technologies.

## Author contributions

F. C. conceived the study, performed most of the experiments, analyzed the results and wrote the paper. X. H. and K. W. revised the manuscript. T. J. and H. F. provided found support and experimental design. All authors read and approved the final manuscript.

## Conflicts of interest

There are no conflicts to declare.

## Supplementary Material

RA-013-D3RA03361A-s001

## References

[cit1] He Y. F., Cheng K., Zhong Z. T., Hou X. L., An C. Z., Zhang L., Chen W., Chen W., Liu B., Yuan L., Zhao Y. D. (2023). Anal. Chim. Acta.

[cit2] Wang W. X., Yang D., Zhou Y. F., Zhang Y. D., Guan L. J., Zhang X. F., Zhang W. M., Xue W. M., Huang S. P. (2022). J. Fluoresc..

[cit3] Li J. B., Liu H. W., Fu T., Wang R., Zhang X. B., Tan W. (2019). Trends Chem..

[cit4] Ding H., Cai Y. J., Chen J. X., Lu T., Wen W. J., Nie G. H., Wang X. J. (2019). Mikrochim. Acta.

[cit5] Fu Y., Gao G., Zhi J. (2019). J. Mater. Chem. B.

[cit6] Markovi Z. M., Labudova M., Danko M., Matijasevic D., Todorovi-Markovi B. (2020). ACS Sustainable Chem. Eng..

[cit7] Li Y., Lin H., Luo C., Wang T., Jiang C., Qi R., Huang R., Travas-sejdic J., Peng H. (2017). RSC Adv..

[cit8] Zhou Z. J., Ushakova E. V., Liu E. S., Bao X., Li D., Zhou D., Tan Z. A., Qu S. N., Rogach A. L. (2020). Nanoscale.

[cit9] Li K., Chen J., Yan Y., Yin Y., Li H., Xi F., Chen P. (2018). Carbon.

[cit10] Zhu Y., Li J. K., Yan Z. H., Zhao N., Yang X. M. (2022). Langmuir.

[cit11] Mohandoss S., Palanisamy S., You S. G., Shim J. J., Lee Y. P. (2021). Anal. Methods.

[cit12] Mohandoss S., Ganesan S., Gamesan S., Palanisamy S., You S. G., Velsankar K., Sudhahar S., Lo H. M., Lee Y. R. (2023). Chemosphere.

[cit13] Sonaimuthu M., Ganesan S., Anand S., Kumar A. J., Palanisamy S., You S. G., Velsankar K., Sudhahar S., Lo H. M., Lee Y. R. (2023). Environ. Res..

[cit14] Mohandoss S., Khanal H. D., Palanisamy S., You S. G., Shin J. J., Lee Y. R. (2022). Anal. Methods.

[cit15] Mohandoss S., Ahmad N., Khan M. R., Velu K. S., Palanisamy S., You S. G., Kumar A. J., Lee Y. R. (2023). Environ. Res..

[cit16] Huang S., Yang E., Liu Y., Yao J., Su W., Xiao Q. (2018). Sens. Actuators, B.

[cit17] Li H. T., He X. D., Kang Z. H., Huang H., Liu Y., Liu J. L., Lian S. Y., Tsang C. H., Yang X. B., Lee S. T. (2010). Angew. Chem., Int. Ed..

[cit18] Ding H., Yu S. B., Wei J. S., Xiong H. M. (2016). ACS Nano.

[cit19] Tong X. Y., Lin X. F., Duan N., Wang Z. P., Wu S. J. (2022). ACS Sens..

[cit20] Song Y. B., Zhu S. J., Zhang S. T., Fu Y., Wang L., Wang X. H., Zhao X. H., Yang B. (2015). J. Mater. Chem. C.

[cit21] Schneider J., Reckmeier C. J., Xiong Y., Seckendorff M., Susha A. S., Kasák P., Rogach A. L. (2017). J. Phys. Chem. C.

[cit22] Reckmeier C. J., Schneider J., Susha A. S., Rogach A. L. (2016). Opt. Express.

[cit23] Hu S., Tian R., Dong Y., Yang J., Liu J., Chang Q. (2013). Nanoscale.

[cit24] Zhu S., Meng Q., Wang L., Zhang J., Song Y., Jin H., Zhang K., Sun H., Wang H., Yang B. (2013). Angew. Chem., Int. Ed..

[cit25] Cao F. J., Wang L., Feng C. L., Lin X., Feng H. (2021). RSC Adv..

[cit26] Yew Y. T., Loo A. H., Sofer Z., Klímová K., Pumera M. (2017). Appl. Mater. Today.

[cit27] Park S., Kim H. (2015). J. Mater. Chem. A.

[cit28] Rein M., Richter N., Parvez K., Feng X., Sachdev H., Klaui M., Mullen K. (2015). ACS Nano.

[cit29] Hola K., Bourlinos A. B., Kozak O., Berka K., Siskova K. M., Havrdova M., Tucek J., Safarova K., Otyepka M., Giannelis E. P., Zboril R. (2014). Carbon.

[cit30] Park S., Kim H. (2015). J. Mater. Chem. A.

[cit31] Guo L., Ge J., Liu W., Niu G., Jia Q., Wang H., Wang P. (2016). Nanoscale.

[cit32] Holá K., Sudolská M., Kalytchuk S., Nachtigallová D., Rogach A. L., Otyepka M., Zbořil R. (2017). ACS Nano.

[cit33] Higgins D., Chen Z., Chen Z. (2011). Electrochim. Acta.

[cit34] liu X., Yu W., Mu X. W., Zhang W., Wang X. H., Gu Q. (2023). Spectrochim. Acta, Part A.

[cit35] Arsalani N., Nezhad-Mokhtari P., Jabbari E. (2019). Artif. Cells, Nanomed., Biotechnol..

[cit36] Wibrianto A., Khairunisa S. Q., Sakti S. C. W., Ni'Mah Y. L., Fahmi M. Z. (2021). RSC Adv..

[cit37] Huang S., Yang E., Liu Y., Yao J., Su W., Xiao Q. (2018). Sens. Actuators, B.

