# Supplementary material for: Facile synthesis of phosphorus and nitrogen co-doped carbon dots with excellent fluorescence emission towards cellular imaging
Source: RSC Adv. 2023 Jul 12;13(30):21088–95. doi: 10.1039/d3ra03361a (PMC10336645; doi:10.1039/d3ra03361a)
Supplement: RA-013-D3RA03361A-s001 [file RA-013-D3RA03361A-s001.pdf]

Electronic Supplementary Material (ESI) for RSC Advances.
This journal is © The Royal Society of Chemistry 2023

Supporting Information

# Facile Synthesis of Phosphorus and Nitrogen Co-doped Carbon Dots with Excellent Emitting Fluorescent towards Cellular Imaging

*Fang-Jun Cao, Xiang Hou, Kai-Feng Wang ,Tie-Zhi Jin*, Hui Feng**

Shaanxi Institute of Zoology, Xi'an, Shaanxi 710072, P. R. China

*Corresponding author: jintiezhi@xab.ac.cn; fenghui84@126.com

Figures S1–S9.

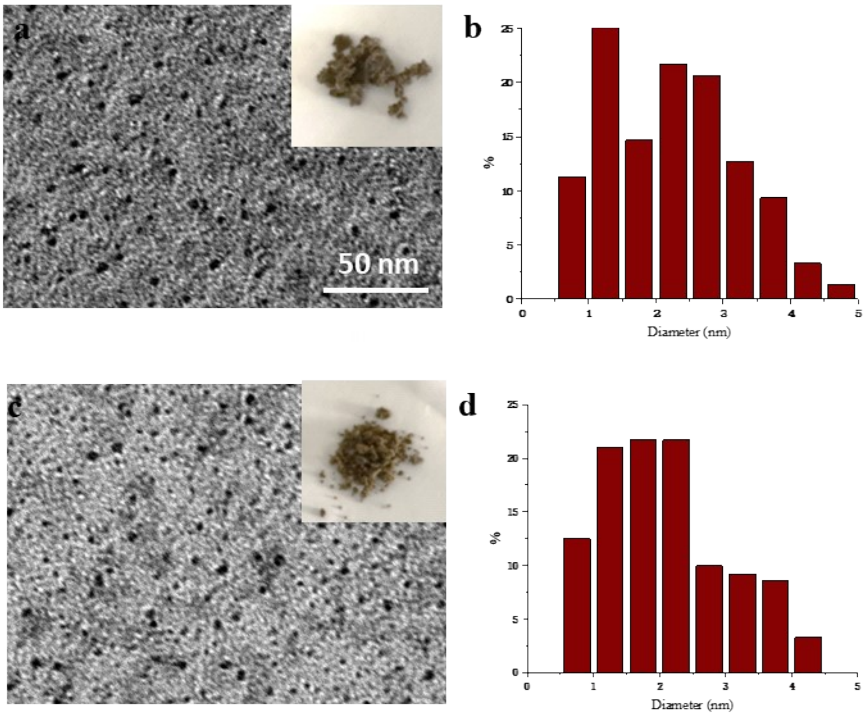


**Figure S1**. (a-b) TEM images and size distribution diagram of as-prepared bCDs; (c-d) TEM images and size distribution diagram of as-prepared gCDs.

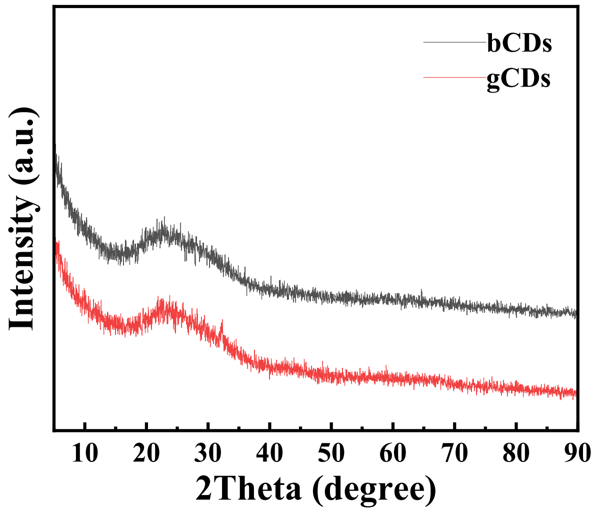


**Figure S2**. XRD pattern of as-prepared bCDs and gCDs.

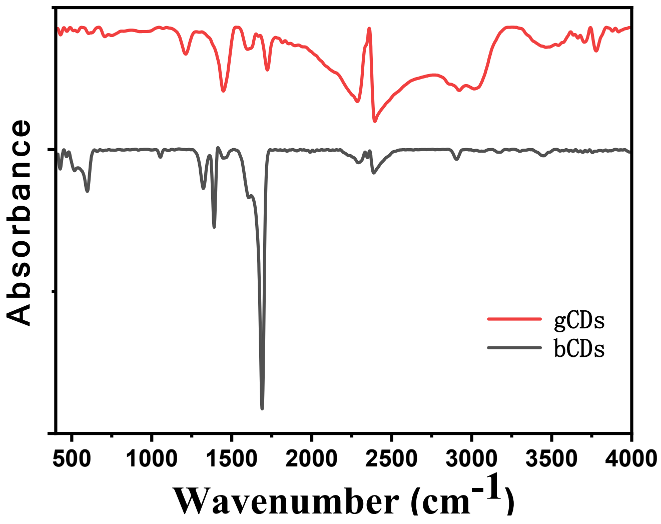


**Figure S3**. FTIR spectra of as-prepared bCDs and gCDs.

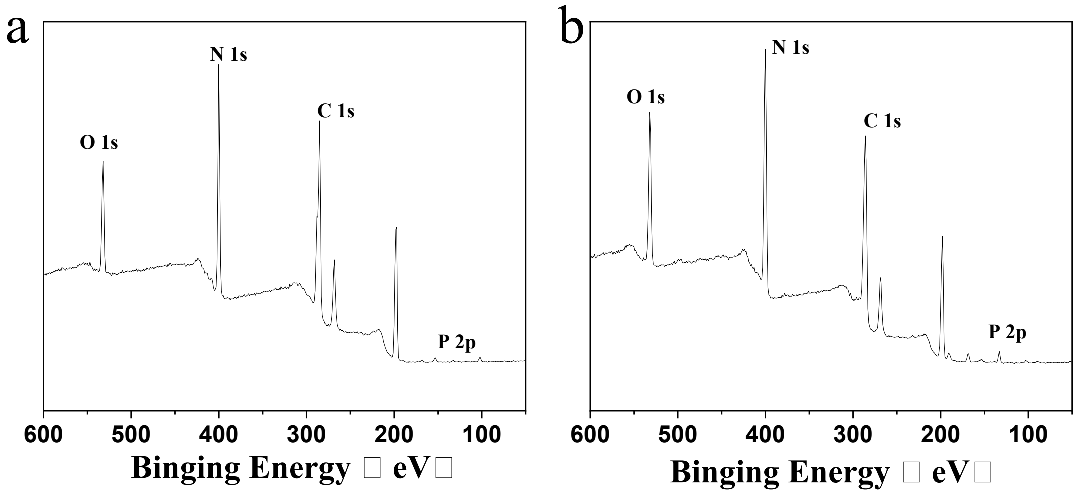


**Figure S4**. The XPS survey spectra of the separated fractions of (a) bCDs and (b) gCDs.

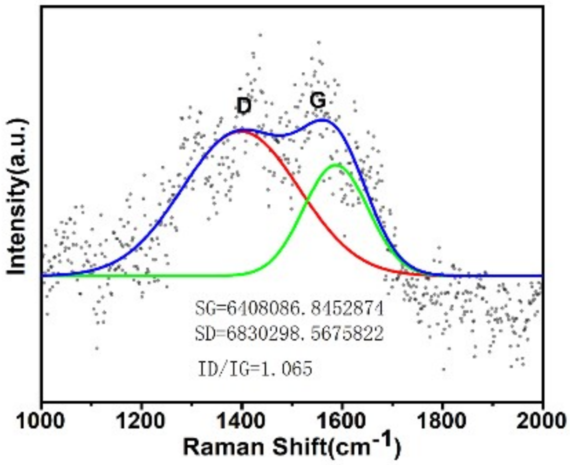


**Figure S5.** Raman spectra of as-prepared bCDs and gCDs.

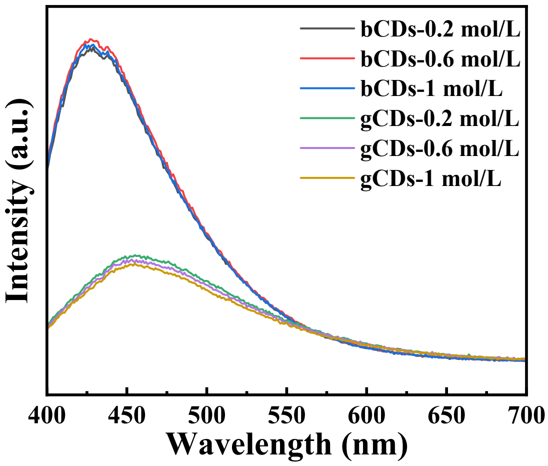


**Figure S6.** Fluorescence emission spectrum of the separated fractions of (a) bCDs, (b) gCD in hydrochloric acid solutions of different concentrations.

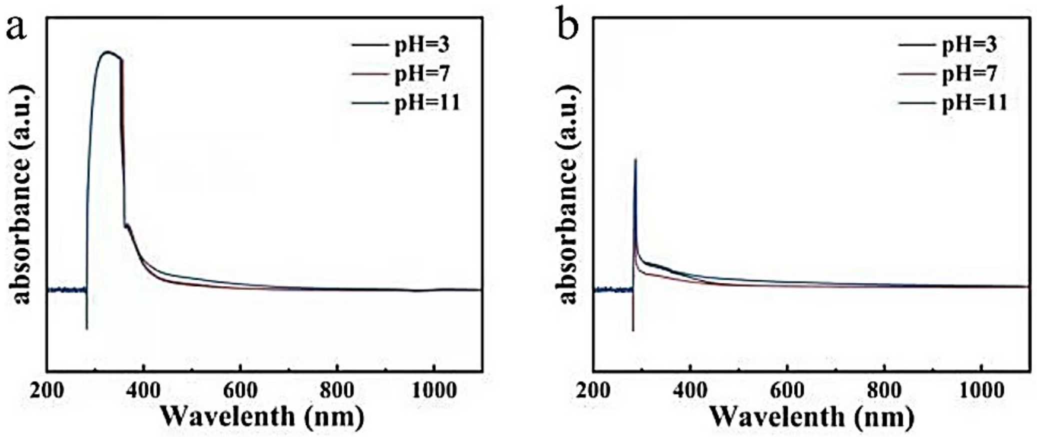


**Figure S7.** Ultraviolet absorption spectrum of the separated fractions of (a) bCDs, (b) gCD in hydrochloric acid solutions of different concentrations.

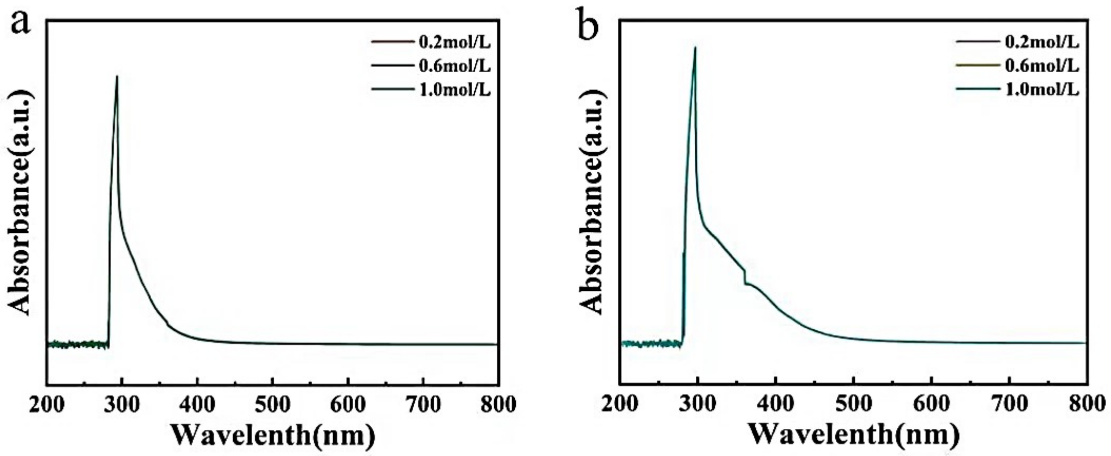


**Figure S8.** Ultraviolet absorption spectrum of the separated fractions of (a) bCDs, (b) gCDs in sodium chloride solutions of different concentrations.

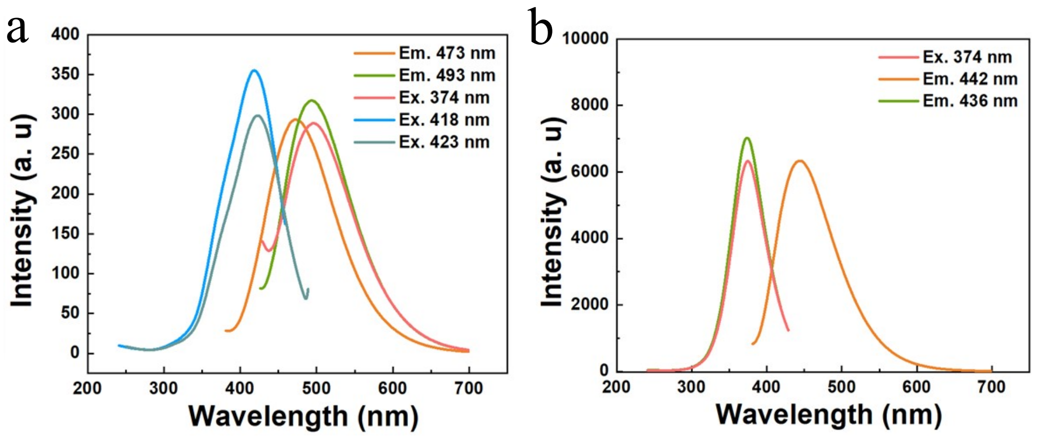


**Figure S9.** Emission spectra of all obtained fractions in water measured at given excitation wavelength: (a) bCDs, (b) gCDs.

**Table S1** Instrument equipments and models

| Instrument Equipment | Model |
|---|---|
| Transmission electron microscopy | G2 F20 S-TWIN |
| HAADF-STEM instrument | Themis Z, FEI |
| Scanning electron microscope | SU8220, Hitachi |
| UV–vis spectrometer | Record S 600 |
| X-ray photoelectron spectroscopy technology | Thermo Fisher Escalab 250X |
| X-ray diffraction | A Bruker D8 ADVANCE diffractometer using Cu Ka radiation |
| Infrared spectrometer | Bruker Tencer 2 |
| Raman spectrometer | HORIBA, HR800 |
| Fluorescence Spectrophotometer | ETT-F97 Series |

**Table S2** Elemental analysis of the as-synthesised bCDs and gCDs:

| Sample | Elemental content (%) | | | | |
|---|---|---|---|---|---|
| | C | H | O | N | P |
| bCDs | 48.13 | 1.04 | 15.81 | 33.76 | 1.26 |
| gCDs | 42.54 | 2.16 | 16.73 | 34.92 | 3.65 |
